# Association of skeletal muscle mass and risk of hypertension in Korean adults: secondary analysis of data from the community-based prospective cohort study

**DOI:** 10.3389/fnut.2023.1254109

**Published:** 2023-11-23

**Authors:** So Young Bu

**Affiliations:** Department of Food and Nutrition, Daegu University, Gyeongsan, Republic of Korea

**Keywords:** skeletal muscle, hypertension, obesity, aging, KoGES

## Abstract

**Background:**

Cross-sectional studies have revealed a link between low muscle mass and hypertension. However, whether the degree of muscle mass predicts hypertension risk has not been confirmed. This study aimed to verify an association between skeletal muscle mass and incident hypertension in a longitudinal follow-up of middle-aged Korean adults.

**Methods:**

The community-based prospective Korean Genome and Epidemiology Study (KoGES) data from 2,669 participants who were free of hypertension at baseline were prospectively assessed at 2-year intervals for 16 years. The participants were divided into tertiles T1–T3 of relative skeletal muscle mass (RSM) according to their baseline whole-body skeletal muscle mass measured as bioelectrical impedance. Incident hypertension was estimated using multivariate logistic regression with the Cox proportional hazard regression model.

**Results:**

Over the 16-year follow-up, the rates of incident hypertension at RSM T1, T2, and T3 were 18.7, 17.1, and 13.4% in men (*P* for trend = 0.0002) and 18.8, 14.7, and 12.9% in women (*P* for trend = 0.0007), respectively. The multivariate adjusted hazard ratios (HRs) with 95% confidence intervals (CIs) for the incidence of hypertension for men and women in T1 and T2 were 1.36 (1.11–1.67) and 1.59 (1.31–1.94), and 1.20 (0.99–1.46) and 1.70 (1.41–2.04), respectively, compared with T3 as the reference.

**Conclusion:**

A low skeletal muscle mass in middle-aged Korean men and women was significantly associated with incident hypertension in later life. Further investigation is needed to comprehend the mechanisms of this relationship and validate the findings in a large cohort.

## Introduction

Hypertension is a major underlying risk factor for cardiovascular illnesses and metabolic syndrome, and the leading contributor to the global health burden (1). Hypertension is a major health concern in Korea, where it affects 6.3 million men and 5.8 million women (2). Risk factors for hypertension include body composition factors such as a high body mass index (BMI), abdominal fat, and low muscle mass (1, 3). A meta-analysis of 35 studies that included 3,219 patients found that a mean BMI reduction of 2.27 kg/m^2^ significantly lowered clinic systolic (SBP) and diastolic (DBP) blood pressure values by 5.79 and 3.36 mmHg, respectively (4). Increased blood pressure also contributes to metabolic syndrome, which is associated with cardiovascular disease, diabetes, its related complications, and mortality (5). As a causal relationship between hypertension and obesity assessed as BMI or body fat content, is a growing concern, it was postulated that low muscle mass or weakness would also be associated with risk of hypertension.

Sarcopenia, defined as decreased muscle mass or strength, is associated with low energy intake and malnutrition (6, 7). Trends in inadequate energy intake and low food intake of high-protein sources during aging have been attributed to skeletal muscle mass and decline, depending on body weight or visceral obesity (7–9). A decline in muscle mass is associated with factors composing metabolic syndrome; insulin resistance, high abdominal fat, and hypertension (10–12). Low muscle mass also increased cardiovascular risk and all-cause mortality (9, 13–15). Decreased skeletal muscle mass is related to arterial stiffness, which precedes the development of hypertension (16) in young adults and patients with diabetes (17, 18). In addition, skeletal muscle has an endocrine function by secreting several myokines (19) and dysregulated crosstalk leads to, or confounds energy and fluid homeostasis (20–22), which might result in hypertension. Recent large-scale observational investigations have revealed an inverse association between skeletal muscle mass and blood pressure (12, 23–25). However, little evidence has substantiated a direct connection between muscle mass and hypertension. For example, a cross-sectional study found that hypertension was independently associated with sarcopenia in men and women, and most sarcopenia-related parameters, including grip strength and chair stand tests, were negatively associated with hypertension (12). However, a lean body mass has been positively associated with blood pressure (26, 27). These conflicting results were due to variations in populations and small study cohorts among studies. In addition, most studies were cross-sectional; thus, they do not support a causal relationship between skeletal muscle mass and hypertension. Although a few longitudinal studies have examined the incidence of hypertension over periods up to 4 years (24, 25), both muscle loss and development of hypertension are time-consuming processes. Therefore, the long-term consequences of low muscle mass from middle age should be further elucidated.

The present study prospectively investigated the incidence of hypertension and aimed to establish causal relationships between muscle mass and hypertension among Korean adults aged between 40 and 60 years by analyzing 16 years of follow-up data generated by the Korean Genome and Epidemiology Study (KoGES).

## Materials and methods

### Study population

**FIGURE 1:**
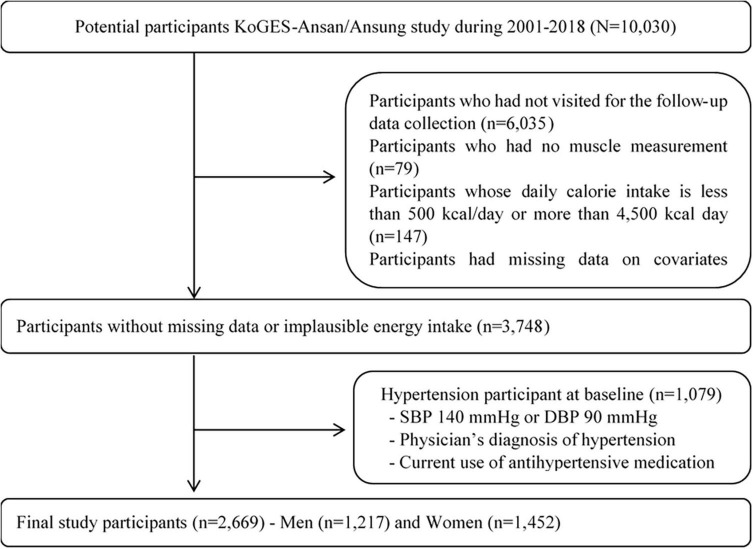
Flow diagram for selection of study participants.

This study is a secondary analysis of the community-based, prospective, KoGES cohort study that has been ongoing since 2001. A total of 10,030 Korean adults aged 40–69 years were enrolled in the KoGES study for a baseline investigation (2001–2002) and genetic analyses, and followed up at 2-year intervals until the end of 2018. All participants provided written informed consent to participate before KoGES started (28). The first and last (8th) follow-up examinations were between 2003–2004 and 2016–2018, respectively. Rates of 1st to 8th follow-up compliance in the Ansan and Ansung cohorts were 80, 81, 65, 65, 61, 62, 59, 57%, respectively, and have served as baseline data (28–30). Figure 1 shows the flow chart of study participants. Among the 10,030 initially enrolled participants, 6,282 were excluded because they did not attend follow-up appointments (*n* = 6,035); absent muscle measurement (*n* = 79), missing data (*n* = 21), and abnormal energy intake (*n* = 147). In addition, 1,079 patients with baseline hypertension were further excluded. Consequently, therefore, data from 2,669 participants were analyzed (Figure 1). The investigators followed ethical guidelines, and all personal information was rendered innominate before data were obtained. Hence, informed consent was not required to participate in this study. The Institutional Review Board of Daegu University (No. 1040621-202303-BR-E004) approved the study protocol.

### Blood pressure measurement and diagnosis of hypertension

Participants SBP and DBP were measured using an automated oscillometric device at least twice in the supine position after a 5 min rest (31), and average blood pressure values in the left and right arms were obtained. Hypertension was defined as SBP > 140 mmHg, DBP > 90 mmHg, or a history of hypertension, and prescribed with antihypertensive medications, (32). At subsequent follow-up examinations, new hypertension onset was defined as SBP and DBP > 140 and > 90 mmHg, respectively, based on clinical assessment data, physician-diagnosed hypertension and or hypertension controlled by antihypertensive medications based on participant answers to a questionnaire.

### Measurement of skeletal muscle and calculation

Skeletal muscle mass was assessed using an InBody 3.0 bioelectrical impedance analyzer (Biospace, Seoul, Korea) (33), the reproducibility and accuracy of which has been validated to measure body composition, muscle mass, and body fat (34, 35), and muscle mass assessed by bioelectrical impedance analysis (BIA) can predict risk of diabetes (33). The relative skeletal muscle mass (RSM) (36), measured by dividing muscle mass by body weight (kg) then multiplying the result by 100, and RSM predicted disease risk better than appendicular muscle mass or height-adjusted appendicular muscle mass (30, 37).

### Clinical and laboratory measurements

All participants were clinically assessed at each follow-up. Body weight was measured within 0.1 kg, and height and waist circumference (cm) were measured according to the standard protocol for Korean CDC (31). The BMI was calculated as body weight (kg)/height (m^2^). The participants fasted for 8 h, then blood was sampled from the antecubital vein. Thereafter, values for serum glucose, triglycerides, total cholesterol, high-sensitivity C-reactive protein (hsCRP), albumin, blood urea nitrogen (BUN), and serum creatinine were analyzed according to the guidelines (31).

### Main covariates

Participants were interviewed and completed questionnaires to obtain their medical history, lifestyle, dietary and demographic information. The main covariates were age, sex, diabetes, hypertension, hyperlipidemia, smoking status, educational level, alcohol consumption, and diet. Total energy and sodium intake were estimated using a food frequency questionnaire (38). Excessive, insufficient, and normal energy intake were defined as > 125, < 75, and 75-125% of the estimated energy requirement (EER), and chronic disease risk reduction intake (CDRR) of the sodium for Koreans (39), is 2,300 mg for adults aged < 65 years, 2,100 mg for adults ≥ 65 years old, respectively, for both men and women. Diabetes was defined as fasting glucose (at least 126 mg/dL, physician-diagnosed diabetes, or using antidiabetic medication). Dyslipidemia was determined as a total cholesterol > 240 mg/dL, or physician-diagnosed dyslipidemia. Obesity status, abdominal obesity, excessive or insufficient energy intake, and CDRR of sodium were defined and categorized in the stratified population. The obesity criterion herein was a BMI ≥ 25 kg/m^2^ for both men and women; this was based on Asian and Pacific findings (40).

### Statistical analyses

The baseline RSM of participants was classified into tertiles (T1–T3). Descriptive statistics were applied to data summary of the participants according to RSM tertiles within the same sex. The data of continuous variables are presented as means and standard errors, and the data of categorical variables were reported as numbers and percentages. Continuous variables were compared among three quartiles using analysis of variance (ANOVA). Categorical variables were compared among tertiles using Chi-square test. The cumulative incidence of hypertension and hazard ratios (HRs) were calculated by constructing Cox proportional hazard regression models and the risk of developing hypertension according to RSM tertiles were separately assessed in men and women. The highest RSM tertile served as a reference. The incidence rate of hypertension was generated by dividing the number of hypertension incidence by the sum of person-years without hypertension during the follow-up period in each RSM tertile. Multivariate hazard regression models were adjusted for main covariates. The covariates adjusted for the multivariate analysis were age, height, educational level, SBP, diabetes or dyslipidemia, hsCRP, current smoking, alcohol consumption, and daily energy intake and sodium intake. Collinearity among variables was determined by assessing variance inflation factors (VIF) and the proportion of variation (PV) > 10 or > 0.5. Hence, waist circumference was not included in Cox proportional hazard regression model because of a high PS value (0.91543). Events during hypertension-free survival estimated from Kaplan–Meier curves, and trends of differences among tertiles were evaluated using log-rank tests. Furthermore, Cox hazard ratio for the risk of hypertension by tertiles of skeletal muscle mass were analyzed by stratifying amounts of energy, and sodium intake, the presence of obesity or abdominal obesity. All data were statistically analyzed using SAS software (9.4; SAS Institute, Cary, NC, USA). Values with *P* < 0.05 were considered statistically significant.

## Results

### Baseline characteristics of participants according to the tertiles of RSM

Data derived from 1,217 men and 1,452 women tracked for 16 years and the incidence of hypertension were evaluated according to the RSM tertiles. In the beginning of the cohort, the mean ages in the lowest tertile in men and women were 49.8 and 50.7 years, respectively (Table 1). In women, those in the lower tertile group were older (*P* < 0.0001). The skeletal muscle mass among the RSM tertiles was not significantly different in women. The trend of differences in skeletal muscle mass among tertiles was significant (*P* = 0.0004) in men; however, the difference between the lowest and highest means was subtle (< 2.5%). There was a statistically significant trend of higher levels of body weight, height, BMI, waist circumference, blood pressure, fasting glucose, triglyceride, total cholesterol, and hsCRP in the lower RSM tertiles in both men and women, indicating worse metabolic characteristics than those in the higher tertiles, whereas serum albumin, BUN, and creatinine levels were not significantly different across the tertiles. Particularly, BMI values in the lowest tertile in both men (26.2 kg/m^2^) and women (27.0 kg/m^2^) were in the range of obesity diagnosis criteria (40). Men in the lower tertile were high likelihood of a lower level of education. Smoking status, alcohol intake, and the presence of hyperlipidemia was not significantly different between the tertiles in either men or women. Presence of diabetes mellitus tended to be reduced in higher tertile compared to that in the lowest tertile in women (*P* = 0.0009).

**TABLE 1:** Characteristics according to RSM tertiles of study participants at baseline.

	Men (*n* = 1,217)				Women (*n* = 1,452)			
	T1 (*n* = 405)	T2 (*n* = 406)	T3 (*n* = 406)	*P*	T1 (*n* = 484)	T2 (*n* = 484)	T3 (*n* = 484)	*P*
RSM (%)	69.7 ± 2.4*	74.6 ± 1.2	79.9 ± 2.7	< 0.0001	60.2 ± 2.5	65.0 ± 1.1	70.9 ± 3.1	< 0.0001
Skeletal muscle mass (kg)	50.7 ± 5.7	50.4 ± 5.8	49.1 ± 5.9	0.0004	38.0 ± 4.3*	38.0 ± 3.9	38.0 ± 4.2	0.9842
Age	49.8 ± 7.6	50.0 ± 7.8	49.8 ± 7.8	0.928	50.7 ± 7.8	49.5 ± 7.5	48.5 ± 7.7	< 0.0001
40 ≤ year < 50	236 (58.3)†	225 (55.4)	242 (59.6)	0.5814	257 (53.1)	286 (59.1)	325 (67.2)	< 0.0001
50 ≤ year < 60	113 (27.9)	113 (27.8)	100 (24.6)		143 (29.6)	136 (28.1)	92 (19.0)	
60 ≤ year < 70	56 (13.8)	68 (16.8)	64 (15.8)		84 (17.3)	62 (12.8)	67 (13.8)	
Body weight (kg)	72.7 ± 8.2	68.0 ± 7.9	61.6 ± 7.8	< 0.0001	63.3 ± 7.9	58.5 ± 6.0	53.7 ± 6.2	< 0.0001
Height (cm)	166.5 ± 5.4	167.2 ± 5.7	168.0 ± 5.7	0.0007	153.0 ± 5.2	155.0 ± 5.1	156.3 ± 5.5	< 0.0001
BMI (kg/m^2^)	26.2 ± 2.2	24.1 ± 2.0	21.8 ± 2.1	< 0.0001	27.0 ± 2.6	24.3 ± 1.9	22.0 ± 1.9	< 0.0001
Waist circumference (cm)	87.7 ± 5.7	83.0 ± 5.4	77.0 ± 5.7	< 0.0001	85.1 ± 8.7	79.9 ± 7.7	74.2 ± 6.7	< 0.0001
SBP (mmHg)	114.8 ± 10.9	114.4 ± 9.8	112.0 ± 10.7	0.022	113.5 ± 11.4	110.5 ± 11.4	109.5 ± 12.0	0.0008
DBP (mmHg)	78.0 ± 7.1	77.0 ± 7.0	75.4 ± 7.8	0.0005	75.1 ± 7.6	73.5 ± 8.0	72.4 ± 8.3	0.0004
Total cholesterol (mg/dL)	198.2 ± 32.4	192.1 ± 33.0	184.0 ± 34.1	< 0.0001	193.4 ± 34.1	187.8 ± 34.3	177.8 ± 30.0	< 0.0001
Triglycerides (mg/dL)	193.7 ± 116.8	179.6 ± 114.9	130.0 ± 71.5	< 0.0001	147.3 ± 89.2	138.3 ± 78.9	113.7 ± 53.4	< 0.0001
Fasting glucose (mg/dL)	90.3 ± 17.8	89.6 ± 28.6	84.6 ± 15.0	0.0002	83.9 ± 15.3	82.5 ± 13.2	80.7 ± 12.3	0.0013
hsCRP (mg/L)	0.26 ± 0.57	0.23 ± 0.37	0.19 ± 0.39	0.101	0.21 ± 0.32	0.17 ± 0.34	0.15 ± 0.26	0.002
Albumin (g/dL)	4.40 ± 0.35	4.35 ± 0.32	4.33 ± 0.32	0.0043	4.16 ± 0.28	4.15 ± 0.25	4.15 ± 0.28	0.9718
BUN (mg/dL)	15.2 ± 3.6	15.0 ± 3.5	15.3 ± 3.8	0.4802	13.6 ± 3.3	13.5 ± 3.3	13.2 ± 3.1	0.1201
Creatinine (mg/dL)	0.97 ± 0.16	0.96 ± 0.17	0.93 ± 0.16	0.0018	0.74 ± 0.10	0.73 ± 0.10	0.73 ± 0.10	0.6156
Diabetes mellitus (%)	17 (4.2)	25 (6.2)	15 (3.7)	0.2144	26 (5.4)	11 (2.3)	7 (1.5)	0.0009
Hypertriglyceridemia (%)	17 (4.2)	13 (3.2)	9 (2.2)	0.2801	7 (1.5)	8 (1.7)	10 (2.1)	0.5346
Education								
Above high school	297 (73.3)	269 (66.3)	243 (59.9)	0.0003	186 (38.4)	192 (39.7)	217 (44.8)	0.0993
Below high school	108 (26.7)	137 (33.7)	163 (40.1)		298 (61.6)	292 (60.3)	267 (55.2)	
Current smoker (%)	173 (42.7)	182 (44.8)	208 (51.2)	0.0404	13 (2.7)	8 (1.7)	10 (2.1)	0.5346
Current alcohol-drinker (%)	295 (72.8)	290 (71.4)	299 (73.7)	0.7732	137 (28.3)	144 (29.8)	133 (27.5)	0.7303

*Means ± S.E., †Frequency (%). Men RSM: T, < 72.68; T2, 72.68 ≤ RSM < 76.79; T3, ≤ 76.79. Women RSM: T1, < 63.17; T2, 63.17 ≤ RSM < 67.01; T3, ≤ 67.01. P-values are from ANOVA for continuous variables and Chi-square test for categorical variables to assess trends in differences among tertiles. RSM, relative skeletal muscle mass; BMI, body mass index; BUN, blood urea nitrogen; DBP, diastolic blood pressure; hsCRP, high-sensitive C-reactive protein; SBP, systolic blood pressure.

### Nutrition intake of participants according to the tertile of RSM

Energy intake status, including total energy intake, amount of carbohydrate, protein, and fat intake and energy intake from alcohol is described in Table 2. No significant difference was found in the amount of total energy and energy-producing nutrients or the ratio of those nutrients to total energy intake among the RSM tertiles. The range of sodium intake across the all three tertiles was in the range of 2,899.7–3,302.3 mg, which was higher than recommended amount by Korean dietary reference intake. Sodium intake among the tertiles was not significantly different between men and women. Energy obtained from alcohol in the lowest tertile was 114.9 kcal and 7.1 kcal for men and women, respectively. Men in the lower RSM tertiles obtained more energy from alcohol consumption than those in the higher tertiles. In addition, the percentage of energy from alcohol consumption was not different among the tertiles in men. No substantial difference in alcohol consumption among the tertiles was observed in women, although the significance of the difference was shown owing to the higher values in the middle tertile.

**TABLE 2:** Nutrition intake status according to RSM tertiles of participants.

	Men (*n* = 1,217)				Women (*n* = 1,452)			
	T1 (*n* = 405)	T2 (*n* = 406)	T3 (*n* = 406)	*P*	T1 (*n* = 484)	T2 (*n* = 484)	T3 (*n* = 484)	*P*
Energy (kcal)	1,988.8 ± 508.4*	2,026.1 ± 543.2	2,005.0 ± 601.4	0.6291	1,903.7 ± 589.3	1,859.2 ± 578.8	1,866.1 ± 605.4	0.4519
Carbohydrate (g)	343.7 ± 88.0	350.0 ± 89.6	347.8 ± 103.4	0.6193	340.3 ± 103.6	332.4 ± 106.3	330.1 ± 106.1	0.2877
Protein (g)	68.4 ± 21.4	69.3 ± 24.5	67.9 ± 24.8	0.6904	63.3 ± 25.7	62.5 ± 22.3	64.1 ± 24.4	0.5954
Fat (g)	35.4 ± 15.8	36.3 ± 17.6	35.5 ± 18.7	0.7219	29.9 ± 16.9	29.0 ± 14.5	30.7 ± 18.1	0.289
Carbohydrate (% Energy)^†^	69.4 ± 6.0	69.6 ± 6.3	69.8 ± 6.6	0.6513	71.9 ± 6.2	71.7 ± 6.3	71.2 ± 7.1	0.2318
Protein (% Energy)	13.7 ± 2.1	13.6 ± 2.3	13.4 ± 2.2	0.2621	13.4 ± 2.2	13.4 ± 2.1	13.4 ± 2.4	0.89
Fat (% Energy)	15.7 ± 4.7	15.7 ± 4.8	15.5 ± 5.2	0.7347	13.7 ± 4.8	13.8 ± 4.9	14.5 ± 5.6	0.0654
Sodium (mg)	3,266.6 ± 1,469.1	3,302.3 ± 1,580.6	3,317.2 ± 1,678.0	0.8958	3,007.3 ± 1,375.8	2,899.7 ± 1,523.0	2,945.2 ± 1,539.2	0.5259
Alcohol intake (kcal)	114.9 ± 173.0	118.5 ± 181.0	113.1 ± 167.7	0.9046	7.1 ± 24.4	10.6 ± 39.0	7.5 ± 31.7	0.1829
Alcohol intake^‡^ (% Energy)	5.8 ± 8.1	6.2 ± 9.3	6.0 ± 9.3	0.8042	0.4 ± 1.7	0.6 ± 2.0	0.4 ± 1.8	0.3294

*Means ± S.E.,

^†^Percent intake from total energy intake, ^‡^Caloric intake from alcohol calculated as g of alcohol intake/day × 7.0 kcal/g of alcohol men RSM: T, < 72.68; T2, 72.68 ≤ RSM < 76.79; T3, ≤ 76.79. Women RSM: T1, < 63.17; T2, 63.17 ≤ RSM < 67.01; T3, ≤ 67.01. P-values are from ANOVA to assess trends in differences among tertiles. RSM, relative skeletal muscle mass.

### Accumulated hypertension incidence according to the tertiles of RSM during the follow-up period

**FIGURE 2:**
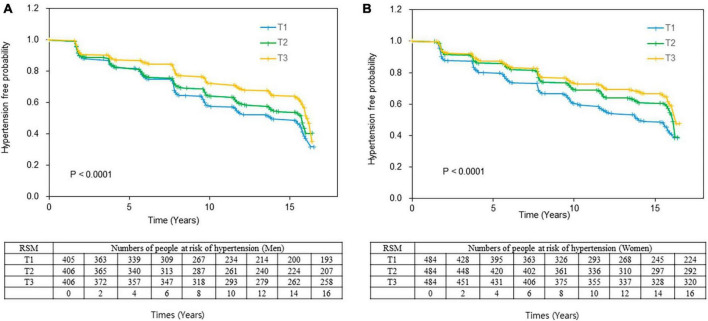
Kaplan–Meier curve of the incidence of hypertension according to RSM tertiles. Event-free rates are significantly associated with skeletal muscle mass, with the worst event-free survival curve for T1 in **(A)** men and **(B)** women. Log-rank tests indicated *P* = 0.0002 and *P* = 0.0005 for men and women, respectively. RSM in men: T1, < 72.68; T2, 72.68 ≤ RSM < 76.79, T3, ≤ 76.79; Women RSM: T1: < 63.17; T2: ≤ 63.17 < 67.01; T3, ≤ 67.01. RSM, relative skeletal muscle mass.

During 16 years of follow-up, 1,280 (47.9%) participants developed hypertension. The total proportions of new hypertension diagnoses were 18.7, 17.1, and 14.0% (*P* for trend = 0.0002) and 18.8, 14.7, and 12.9% (*P* for trend < 0.0001) in men and women in the T1, T2, and T3 tertiles, respectively (Table 3). The Kaplan–Meier curves showed a significantly higher incident probability of hypertension in men and women in the lower, than the higher tertiles (*P* < 0.0001 for both; Figures 2A, B). The probability of incident hypertension was significantly higher in the lower tertiles in participants subgrouped by total energy intake as deficient (< 75%), normal 7 (5–125%), or excessive (≥ 125%) EER and by sodium intake (< 2,300 vs. ≥ 2,300 mg) and abdominal obesity. However, the risk of hypertension did not significantly differ between obese (BMI ≥ 25.0) and non-obese (BMI < 25.0) participants (data are not shown).

**TABLE 3:** Number of hypertension incidents during 16-year follow-up.

Events	*N* (%)			
	T1	T2	T3	*P*
**Total participants (*n* = 2,669)**				
Total hypertension events, *n* (%)	501 (18.8)^†^	422 (15.8)	357 (13.4)	< 0.0001
**Men (*n* = 1,217)**				
Total hypertension events, *n* (%)	228 (18.7)	208 (17.1)	170 (14.0)	0.0002
**Women (*n* = 1,452)**				
Total hypertension events, *n* (%)	273 (18.8)	214 (14.7)	187 (12.9)	< 0.0001

^†^Frequency (%), men RSM: T, < 72.68; T2, 72.68 ≤ RSM < 76.79; T3, ≤ 76.79. Women RSM: T1, < 63.17; T2, 63.17 ≤ RSM < 67.01; T3, ≤ 67.01. P-values are from Chi-square tests of categorical variables to assess trends of differences among tertiles.

### Relationship between RSM and risk of hypertension in Cox hazard regression models

Table 4 shows the results from multivariable Cox hazard regression models used to determine the risk of hypertension according to RSM tertiles. Overall, hypertension risk increased in the lower RSM tertile. Crude HRs (95% CI) for incident hypertension without adjustment (Model I) in T1 and T2 vs. T3 were, respectively, 1.36 (1.11–1.67) vs. 1.59 (1.31–1.94) in men and 1.20 (0.99–1.46) vs. 1.70 (1.41–2.04) in women (*P* for trend < 0.0001 for both). The degree of hypertension risk from lowest to highest RSM tertiles remained similar after a gradual adjustment of age, height and SBP (Model II), education level, smoking and alcohol consumption, total energy intake and sodium intake (Model III), and history of diabetes or hyperlipidemia (Model IV) in various regression models.

**TABLE 4:** Relationship between RSM tertiles and hypertension incidence during 16-year follow-up.

	Model I	Model II	Model III	Model IV
Men				
T3	Ref.	Ref.	Ref.	Ref.
T2	1.36 (1.11, 1.67)**	1.26 (1.03, 1.55)*	1.31 (1.07, 1.61)**	1.32 (1.07, 1.62)**
T1	1.59 (1.31, 1.94)***	1.41 (1.16, 1.73)**	1.51 (1.23, 1.85)***	1.53 (1.24, 1.87)***
*P* for trend	< 0.0001	0.0032	0.0004	0.0003
Women				
T3	Ref.	Ref.	Ref.	Ref.
T2	1.20 (0.99, 1.46)	1.15 (0.94, 1.40)	1.14 (0.93, 1.30)	1.12 (0.92, 1.37)
T1	1.70 (1.41, 2.04)***	1.28 (1.06, 1.55)*	1.28 (1.05, 1.55)*	1.26 (1.04, 1.53)*
*P* for trend	< 0.0001	0.0387	0.0446	0.0612

Data are shown as hazard ratios with 95% confidence intervals. Model I, crude mode; Model II, adjusted for age, sex, and height, SBP; Model III, Model II + education + smoking status + total energy intake + sodium intake + alcohol intake; Model IV; Model III + diabetes mellitus + hyperlipidemia. Men RSM: T, < 72.68; T2, 72.68 ≤ RSM < 76.79; T3, ≤ 76.79. Women RSM: T1, < 63.17; T2, 63.17 ≤ RSM < 67.01; T3, ≤ 67.01. RSM, relative skeletal muscle mass, *< 0.05, **< 0.01, ***p < 0.0001 compared to reference group (T3 within same sex).

**FIGURE 3:**
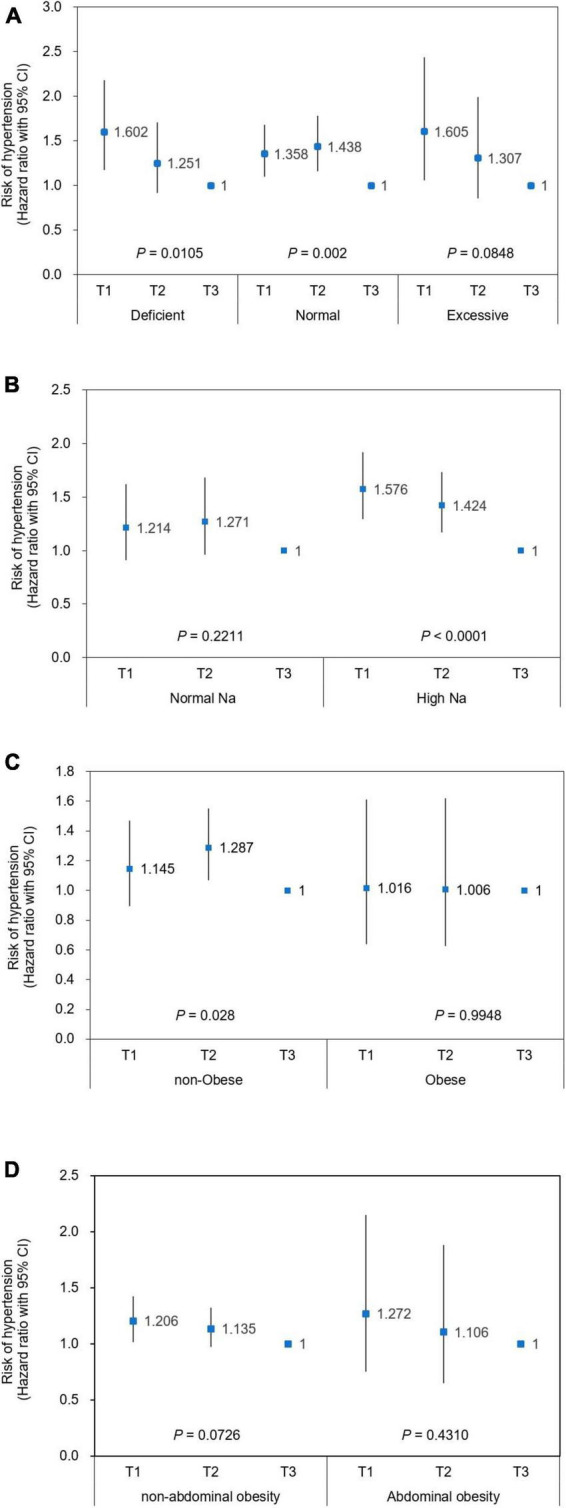
Risk of incident hypertension according to relative skeletal muscle mass in stratified population. Cox hazard ratios (HRs) with 95% CI for the risk of hypertension are plotted according to relative skeletal muscle mass (RSM) in stratified population using **(A)** total daily energy intake, **(B)** sodium intake, **(C)** obesity, and **(D)** abdominal obesity. Men RSM: T1, < 72.68; T2, 72.68 ≤ RSM < 76.79; T3: 76.79 ≤ RSM. Women RSM: T1: < 63.17; T2: ≤ 63.17 < 67.01; T3, ≤ 67.01. Covariates adjusted for Cox proportional hazard model were age, sex, height, level of education, smoking status, alcohol intake, diabetes mellitus, hyperlipidemia at baseline, and SBP, diabetes mellitus, and hyperlipidemia. Criteria for stratification; Intake (% EER): deficient, < 75%; normal, 75–125%; excessive: > 125%. Normal Na: intake below sodium CDRR (2,300 and 2,100 mg at age < 65 and ≥ 65 years, respectively), high Na: intake exceeds sodium CDRR; non-obese, BMI < 25 kg/m^2^; obese, BMI ≥ 25 kg/m^2^. CI, confidence interval; EER, estimated energy requirement; Na, sodium. RSM, relative skeletal muscle mass.

Participants were stratified by selected subgroups and the association between low muscle mass and the risk of hypertension according to RSM tertiles was further explored (Figure 3). An inverse relationship between RSM and risk of hypertension was significant with a similar degree across subgroups by the status of total energy intake (Figure 3A). Sodium intake was associated with a risk of incident hypertension in the lower RSM tertiles that was up to 1.58-fold that of the highest tertile (*P* < 0.0001), whereas the significance of the association disappeared in the group with normal sodium intake (Figure 3B). The association between low RSM and risk of hypertension was significant in the non-obese group (*P* = 0.028), but this disappeared in the obese group (Figure 3C). The trend of association between low RSM and the incident hypertension did not significantly differ between participants with and without abdominal obesity (Figure 3D).

## Discussion

This is the first longitudinal follow-up study to investigate the relationship between muscle mass and hypertension incidence in Korean men and women. We found that low skeletal muscle mass was closely associated with risk of developing hypertension in middle-aged and older Korean adults. Furthermore, this association persisted after adjusting for various covariates.

Recent findings of the Korea National Health and Nutrition Examination Survey (KNHANES) have associated sarcopenia assessed using dual energy X-ray absorptiometry (DXA) with risk of hypertension in an older Korean population; the odds ratios (ORs) for having hypertension were 3-fold higher in patients with sarcopenia compared with those who did not have sarcopenia and were not obese (23). Sarcopenia assessed using a BIA is associated with the incidence of metabolic syndrome and hypertension in older Korean men and women (41, 42). The present study associated a decrease in RSM determined by BIA with an increased incidence in hypertension during 16 years of follow-up. These results agree with, and add to the findings of a large prospective longitudinal study of hypertension risk in Koreans who were evaluated for up to 4 years based on health check-up data. That study associated a low skeletal muscle index with increased blood pressure, and an increase in the number of patients who were prescribed with antihypertensive medications (24). The longitudinal Tobago Health Study of men with African ancestry for 6 years, found that skeletal muscle loss was related to a risk of hypertension (43). However, skeletal muscle mass in that study was not directly assessed, but was indirectly estimated by measuring intermuscular adipose tissue. Results regarding an association between skeletal muscle mass and high blood pressure are contradictory. Increased lean body mass (LBM) is concurrent with increased blood pressure in adolescents and adults (26). However, LBM was indirectly estimated using formula-based values from skinfold and weight measurements. Another cross-sectional study identified an association between a high LBM and decreased blood pressure in adults aged 20–35 years (27). However, corroborating a causal relationship between muscle mass and hypertension in these studies was limited in terms of study design, age, and numbers of participants. To the extent that we are aware, this long-term follow-up study suggests a relationship between skeletal muscle mass and hypertension. At 16 years of follow-up, 5.4% of the participants were newly diagnosed with hypertension. When the participants were categorized according to tertiles of RSM, the overall frequency of incident hypertension was 18.9, 17.7, and 13.4 in men and 17.5, 15.7, and 12.9 in women for T1, T2, and T3, respectively. The results of the present, and a previous longitudinal study have significantly advanced understanding of the consequences of low muscle mass in the development of hypertension.

The association between decreased muscle mass and incident hypertension was stronger in participants in the lowest RSM tertile and was independent of the total energy intake in the present study. When the Cox hazard model was tested in the stratification of energy intake level (< 75% EER, adequate, and excessive intake), the energy intake did not affect the RSM-associated incidence of hypertension. However, when the study population was stratified by obesity according to BMI, the significance of the association remained and disappeared in non-obese and obese participants, respectively, indicating that a higher vs. lower BMI is an independent component for developing hypertension (4, 5). Nevertheless, the association between decreased RSM and risk of hypertension in the subgroup of excessive energy intake was significant. Therefore, the effects of obesity and related factors cannot be concluded from our findings. Risk of diabetes is increased in non-obese (BMI < 25 kg/m^2^) persons with the lowest, compared with the highest muscle mass (30). The present findings were similar, in that only hypertension risk was increased only in non-obese participants. This result is meaningful because Asian populations, including Koreans, tend to have a relatively high ratio of insulin resistance despite a low BMI, but the degree of skeletal muscle mass and body fat % predicted risk of metabolic syndrome, including hypertension beyond categories by BMI (5, 44).

Besides obesity, sodium intake is thought to be involved in the occurrence of hypertension, in line with high energy intake and accumulation (1, 2, 7). Koreans intake relatively high amounts of sodium, and a recent factsheet suggests that ∼50% of the population consumes > 4,000 mg/day, which is twice the intake goal of the Korean dietary reference intake (KDRI) (39). According to a study published in 2013, the mean sodium intake of Korean adults ≥ 30 years increased from 4,464 to 4,831 mg/day, which is consistent with an increase in the prevalence of hypertension from 24.5 to 26.9% (45). The population of this study was stratified based on sodium intake (< 2,300 and ≥ 2,300 mg/day), which is the reference value for increased risk of chronic disease (39), and found a significant association between RSM and the incidence of hypertension (*P* < 0.0001) in the population with higher sodium intake, whereas the association disappeared in the population with adequate sodium intake. The results indicated that preserving of muscle mass could be a modifying factor for hypertension in populations that typically have a higher sodium intake than those who comply with the recommendation. In addition, the association between RSM and hypertension did not differ regardless in subgroups based on total daily energy intake. This result might be associated with the absence of changes in total daily energy intake or fat intake among RSM tertiles. High energy intake including fat, which is directly linked to obesity, leads to hypertension (46). The consequences of hypertension incidence vary according to the intake fat that mainly comprises saturated fatty acids, monounsaturated fatty acids, and polyunsaturated fatty acids including omega-3 fatty acids (47, 48). For example, dietary intake of SFAs and hypertension closely correlate in women and middle-aged and older adults according to the cross-sectional analysis of the National Health and Nutrition Examination Survey (47). The intake of SFA and trans fats significantly increased incident hypertension over 12.9 years of follow-up according to the Women’s Health Study (WHS) in the USA (48). This suggested that types of fatty acids are important in the development of hypertension. This study could not analyze the types of fat intake in participants because information about types of fat in their diet was not available. The obese group who typically consume fat that might include various type of fatty acids. However, a significant relationship was not found between RSM and the risk of hypertension in this group. Types of fat might have affected the results. The intake of at least saturated fat vs. unsaturated fat should be investigated in a future study.

Bioelectrical impedance analysis is a relatively simple and non-invasive method of assessing muscle content in large populations. Several factors such as hydration status, exercise intensity, and degree of mineralization of fat-free tissue can affect BIA results (49, 50). Thus, potential measurement error should be cautiously avoided. Although hydration status of participants was not precisely reflected in BIA data in the study, the results from the original KoGES cohort study showed that skeletal muscle mass assessed by BIA increased the incidence of diabetes (33). In addition, the study participants were categorized according to the range (tertile) of skeletal muscle mass, the range of skeletal muscle within each tertile (5.7 and 4.2 kg for men and women, respectively). This covered reported error ranges due to hydration (1.5-5.0 kg) (51, 52). Another point is that BIA alone might not be as competent to assess skeletal muscle mass compared with other tools such as DXA and computed tomography. However, a sufficient accumulation of data supports the validity of BIA in the assessment of appendicular skeletal muscle (33–35, 49, 53). The results of BIA were compatible with those assessed by DXA through the age range of the adult population (49, 53). In patients with diabetes, the correlation coefficient between BIA and DXA was 0.981 (30). The BIA results of scanned whole-body and calculated appendicular muscle mass closely correlated (34, 35). These results suggested that BIA provided a reasonable estimate of skeletal muscle in the present study. Nevertheless, the data of this study might require validation with other reliable methods assessing skeletal muscle mass. Future studies should measure body composition using DXA or computed tomography for comprehensive assessments. This would render study’s findings applicable in predicting disease risk and developing strategies for securing muscle mass at the community level.

Low skeletal muscle mass might lead metabolic disorders and disabilities in physical performance. Age-related changes in body composition and their relationships are associated with disease processes, mainly in adults aged ≥ 60 years. For example, the Health, Aging, and Body Composition Study of older adults associated the incidence of type 2 diabetes with muscle weakness and decreased muscle mass over a 6-year follow-up (54) whereas the KNHANES study found a concordant relationship between low muscle mass and hypertension in participants ≥ 60 years (23). Moreover, a recent longitudinal study of Korean adults aged 20-80 years found a significant inverse relationship between skeletal muscle mass or the degree of muscle change over time and incident metabolic syndrome (41). These results suggested that a risk of metabolic disease is associated with low muscle mass in the entire age group and the older population. The present study of community-dwelling Korean adults aged 40–70 years, of whom ∼ 60.5% were aged 40–50 years revealed a significant association between low muscle mass and incident hypertension. Collectively, results of this study highlighted the importance of securing and increasing skeletal muscle mass in younger adults.

Although this study showed a similar pattern of relationship between RSM and incident hypertension in men and women, the significance of this association seemed slightly stronger in men than in women. However, a sex-based connection between skeletal muscle mass and blood pressure reported in previous studies is not certain. Patterns of muscle loss might differ between men and women as they age. Skeletal muscle mass peaks between the 30s and 40s, then men start to lose muscle mass in their 70s, whereas women do so in their 40s (55). Sex hormones affect skeletal muscle regeneration and hypertension (56–58). When estrogen levels decrease, such as during menopause, body fat increases and skeletal muscle mass tends to decline (59). Estrogen confers protective effects against cardiovascular disease including hypertension (58), the prevalence of hypertension increases in women more than in men after the age of 50 years, which is an approximate timeline of estrogen loss (57). A recent cohort study found that an inverse relationship between low muscle mass and hypertension is significant only in men (24), indicating that men with a low muscle mass tend to develop hypertension more easily than women. However, in this study, a significant association between the tertiles of muscle mass and hypertension was maintained in women after adjustment for menopausal status (data not shown). Additional studies validating this association in a homogeneous menopausal population might provide a possible explanation.

The mechanisms(s) underlying the relationship between low muscle mass and hypertension are not fully understood. Initially, low skeletal muscle mass lead to insulin resistance, because insulin-mediated blood glucose is mostly utilized in muscle tissue (37). Insulin resistance has been considered as a main risk factor for hypertension (60). Hence, under insulin resistance, diminished skeletal muscle has decreased capacity to uptake plasma energy substrates and lower the glucose utilization process, and consequently blood vessels must bear high glucose, which might disturb the balance of blood flow (61). In addition, low-grade inflammation and oxidative stress due to conditions that induce insulin resistance might be related to hypertension (20). Myokines produced by skeletal muscle, such as irisin, which are affected by physical activity, potentially mediate the increase of blood pressure. For example, a gene variant of irisin is associated with hypertension, and increased irisin levels are associated with high blood pressure and the prevalence of hypertension-related stroke (21). Low skeletal muscle mass is related to increased arterial stiffness and might also mediate hypertension due to sarcopenia (62–64).

This study included a cohort of Korean community residents, which might limit extrapolating the data to the general Korean population. In addition, body composition varies by region and the cohort were single rural and urban dwellers. Hence, generalizing study results to larger populations would be difficult. Future studies need to include participants from various regions and a larger cohort. Exercise-related factors were not included in the analysis of this study. All of participants were physically active when assessed using the metabolic equivalent task classification, and only two participants exercised regularly more than once per week for at least 30 min. A meta-analysis of 391 randomized-controlled, trial intervention studies (43) showed that exercise has an effect comparable to that of antihypertensive medication in lowering SBP in hypertensive populations. Positive effects of exercise on blood pressure are related to vascular system remodeling and enhanced cardiac function (65, 66). Owing to the limitation of the study population, who dwelled in the same geological area, and a limited number of participants who exercised regularly, this study could not appropriately assess the modifying effect of exercise on the relationship between skeletal muscle mass and hypertension. A study of a large heterogeneous population and various lifestyles that allow subcategorization based on physical activity might clarify this issue.

In conclusion, this community-based prospective cohort study of adult Korean participants significantly associated a low RSM at baseline with incident hypertension during a 16-year follow-up. The results of this study showed that low muscle mass earlier in life could be an independent risk factor for hypertension in Korean adults. An association between muscle mass and hypertension incidence has been identified in a large cohort, and a hypertension prediction model has been examined based on data from the Ansan-Ansung community cohorts (29), which are the same community cohort as the current study. That study focused on factors in the Framingham model used to predict hypertension. This study emphasized the significance of skeletal muscle mass in predicting hypertension risk, which has not been considered in previous Ansan-Ansung community cohorts from the KoGES. Data from this study of Koreans including middle-aged adults, suggest that securing skeletal muscle mass at an earlier age will help to prevent hypertension during aging.

## Data availability statement

The data analyzed in this study is subject to the following licenses/restrictions: the data presented in this study are available on request from the corresponding author upon reasonable request. The data are not publicly available due to privacy protection of people who participated in the KoGES cohort. Requests to access these datasets should be directed to busy@daegu.ac.kr.

## Ethics statement

The studies involving humans were approved by the Institutional Review Board of Daegu University. The studies were conducted in accordance with the local legislation and institutional requirements. The Ethics Committee/Institutional Review Board waived the requirement of written informed consent for participation from the participants or the participants’ legal guardians/next of kin because this is the secondary analysis of the data.

## Author contributions

SB: Conceptualization, Formal analysis, Investigation, Methodology, Project administration, Writing – original draft, Writing – review & editing.
